# Novel Partial Exon 51 Deletion in the Duchenne Muscular Dystrophy Gene Identified *via* Whole Exome Sequencing and Long-Read Whole-Genome Sequencing

**DOI:** 10.3389/fgene.2021.762987

**Published:** 2021-11-26

**Authors:** Qianqian Li, Zhanni Chen, Hui Xiong, Ranran Li, Chenguang Yu, Jingjing Meng, Panlai Shi, Xiangdong Kong

**Affiliations:** ^1^ Genetics and Prenatal Diagnosis Center, Department of Obstetrics and Gynecology, The First Affiliated Hospital of Zhengzhou University, Zhengzhou, China; ^2^ Genokon Institute of Medical Science and Laboratory, Xiamen, China; ^3^ School of Life Science and Technology, Xinxiang Medical University, Xinxiang, China; ^4^ Key Laboratory of Molecular Biophysics of the Ministry of Education, Cardio-X Center, College of Life Science and Technology and Center for Human Genome Research, Huazhong University of Science and Technology, Wuhan, China

**Keywords:** Duchenne muscular dystrophy, partial exonic deletion, breakpoints, whole exome sequencing, long-read whole-genome sequencing

## Abstract

Duchenne muscular dystrophy (DMD), one of the most common progressive and severely disabling neuromuscular diseases in children, can be largely attributed to the loss of function of the *DMD* gene on chromosome Xp21.2-p21.1. This paper describes the case of a 10-year-old boy diagnosed with DMD. Whole exome sequencing confirmed the hypothesized large partial exonic deletion of c.7310-11543_7359del (chrX:g.31792260_31803852del) spanning exon 51 and intron 50 in *DMD*. This large deletion was verified to be *de novo* by PCR, and the two breakpoints were further confirmed by Sanger sequencing and long-read whole-genome sequencing. Notably, this partial exonic deletion was the only complex variation in the deep intron regions or intron–exon junction regions in *DMD*. In addition, the case study demonstrates the clinical importance of using multiple molecular genetic testing methods for the diagnosis of rare diseases.

## Introduction

Duchenne muscular dystrophy (DMD, MIM# 310200) is inherited in an X-linked recessive manner, occurring in 1/3,600 to 1/6,000 live male births (Bushby et al., 2010). DMD is associated with the *DMD* gene (OMIM* 300377), which consists of 79 exons and is one of the largest genes. *DMD* is located on chromosome Xp21.2-p21.1. About two-thirds of patients inherit DMD maternally, while the remaining cases are a result of *de novo* mutations in *DMD* (Lane et al., 1983). There are many *DMD* variants, including those with deletions (∼60%) or duplication (∼7%) of one or more exons, small insertions and deletions (INDELs) within an exon (∼7%), single nucleotide variants (SNVs) (∼20%), and rare mutations such as splice site or intronic mutations (<1%) (Aartsma-Rus et al., 2006) and partial exonic deletions (∼5%). In 2018, Liu et al. observed a novel mutation of c.6241_c.6290 + 1109del1159insAC using targeted next-generation sequencing (NGS). This mutation was a 1,159-bp deletion spanning the last 50 bp of exon 43 and the first 1,109 bp of intron 43, causing the partial deletion of exon 43 of *DMD* (Liu et al., 2018). In 2021, Geng et al. detected intron 44 deletion breakpoints using long-read whole-genome sequencing (LR-WGS). This study was the first to use LR-WGS to explore the possible mechanisms underlying exon deletions starting from intron 44 of *DMD* (Geng et al., 2021). Later in 2021, Chin et al. used LR-WGS to confirm that a 33-year-old G2P1 proband at 26 weeks of pregnancy with a heterozygous 426.1-kb duplication on chromosome Xp21.2 (chX:30936321–31362374, GRCh37/hg19) carried the duplication at chrX:30939526–31362638 as a direct repeat inserted downstream of *DMD* (Chin et al., 2021). In this study, we identified a *de novo* partial exon 51 deletion in *DMD* in a 10-year-old boy diagnosed with DMD by using whole exome sequencing (WES), Sanger sequencing, and LR-WGS.

## Materials and Methods

### Ethics Statement

The family provided written informed consent, and this study was approved by the appropriate local institutional review boards on human subject research at the First Affiliated Hospital of Zhengzhou University.

### Quantitative Fluorescent PCR

The genetic relationship of the proband and the parents was confirmed by quantitative fluorescent polymerase chain reaction (QF-PCR) using the Goldeneye DNA ID System 20A Kit (Peoplespot, Beijing, China).

### Multiplex Ligation-dependent Probe Amplification

A multiplex ligation-dependent probe amplification (MLPA) assay was performed using the SALSA MLPA Kit P034/P035 for *DMD* (MRC-Holland, Amsterdam, Netherlands).

### Whole Exome Sequencing

WES was performed using Illumina library construction and capture kits (Illumina, San Diego, California, USA) according to the manufacturer’s instructions. Briefly, genomic DNA was fragmented with enrichment Bead-Linked Transposomes (eBLT) in Tagmentation Buffer 1 (TB1) (Illumina), with a target fragment size of 200 to 300 bp. The fragment ends were repaired, and an adenosine residue was added to the 3′ end of the fragments. Adaptors were then ligated to the fragments using the Fast DNA Library Prep Set for Illumina CW3045M (CWBIO Inc., China). Exon-containing libraries were captured using IDT xGen exome baits (Integrated DNA Technologies, Inc., USA), and quality and purification were assessed using Qubit 4.0 (Thermo Fisher Scientific Inc., USA). Also, 150 bp pair-end sequencing was conducted on NovaSeq 6000 for sequencing depths greater than 100**×**.

After sequencing, paired-end reads were trimmed using Trimmomatic version 0.36 (Bolger et al., 2014) to filter out low-quality reads and adaptors from the dataset. High-quality reads were aligned to the human reference genome GRCh37/hg19 using Burrows–Wheeler Aligner MEM version 0.7.17 (Li and Durbin, 2009), and the duplicates were removed using Picard version 2.9.0 (http://broadinstitute.github.io/picard). Small variants were identified using the Genome Analysis Toolkit (GATK) version 3.8 (McKenna et al., 2010). Copy number variants (CNVs) were detected by comparing the coverage of depth between the target sample and a baseline, which was determined using other male control samples in the same pipeline. In addition, the breakpoints of CNVs were called in the patient’s BAM file by detecting soft-clipped and abnormal mapping orientation reads. The script for calling CNVs was run using the R programming language, and details are described in Supplementary File 1.

### PCR and Sanger Sequencing

The breakpoints of the partial exonic deletion were confirmed by PCR and Sanger sequencing of DNA samples from the proband, the proband’s mother, and a healthy man (a control subject unrelated to this family without any muscular diseases). The primers used for sequencing are listed in Supplementary Table S1. Primers were designed using Primer-BLAST and were based on the combination of sequences on both sides of the deletion. The PCR product of the mutant type was approximately 1 kb, while that of the wild type was approximately 12 kb. Lastly, the sequences were aligned to the human reference genome sequence (GRCh37/hg19) using BLAST version 2.9.0 (Camacho et al., 2009) to identify the breakpoints of the large deletion.

### Long-read Whole-genome Sequencing

The genomic DNA extracted from the fresh peripheral blood of the proband was purified with AMPure XP magnetic beads (Beckman Coulter, CA, USA) and electrophoresed to confirm its integrity. The concentration of the DNA was 44.0 ng μl^−1^ when measured using Nanodrop 2000 (Thermo, Massachusetts, USA) and 43.4 ng μl^−1^ when measured using Qubit 4.0. The DNA input mass for the single library preparation without topping-up or refuel was 2.0398 μg. LR-WGS was carried out without fragmentation using the SQK-LSK109 kit (ONT, Oxford, UK) on MinION (ONT) with R9.4 flow cells (#FLO-MIN106, ONT) according to the manufacturer’s instructions (GDE_9603_v109_revW_Aug 14, 2019) for 72 h. Raw fast5 data were basecalled using Guppy GPU (version 4.3.4) with a high-accuracy base calling model. After base calling, the sequencing data were aligned to the GRCh37/hg19 reference genome using minimap2 (version 2.17-r941) with “map-ont” model, and structural variants (SVs) calling was performed using sniffles (version 1.0.11) with the option “-s 1 -r 1,000 -q 20”.

## Results

### Clinical Report

**FIGURE 1:**
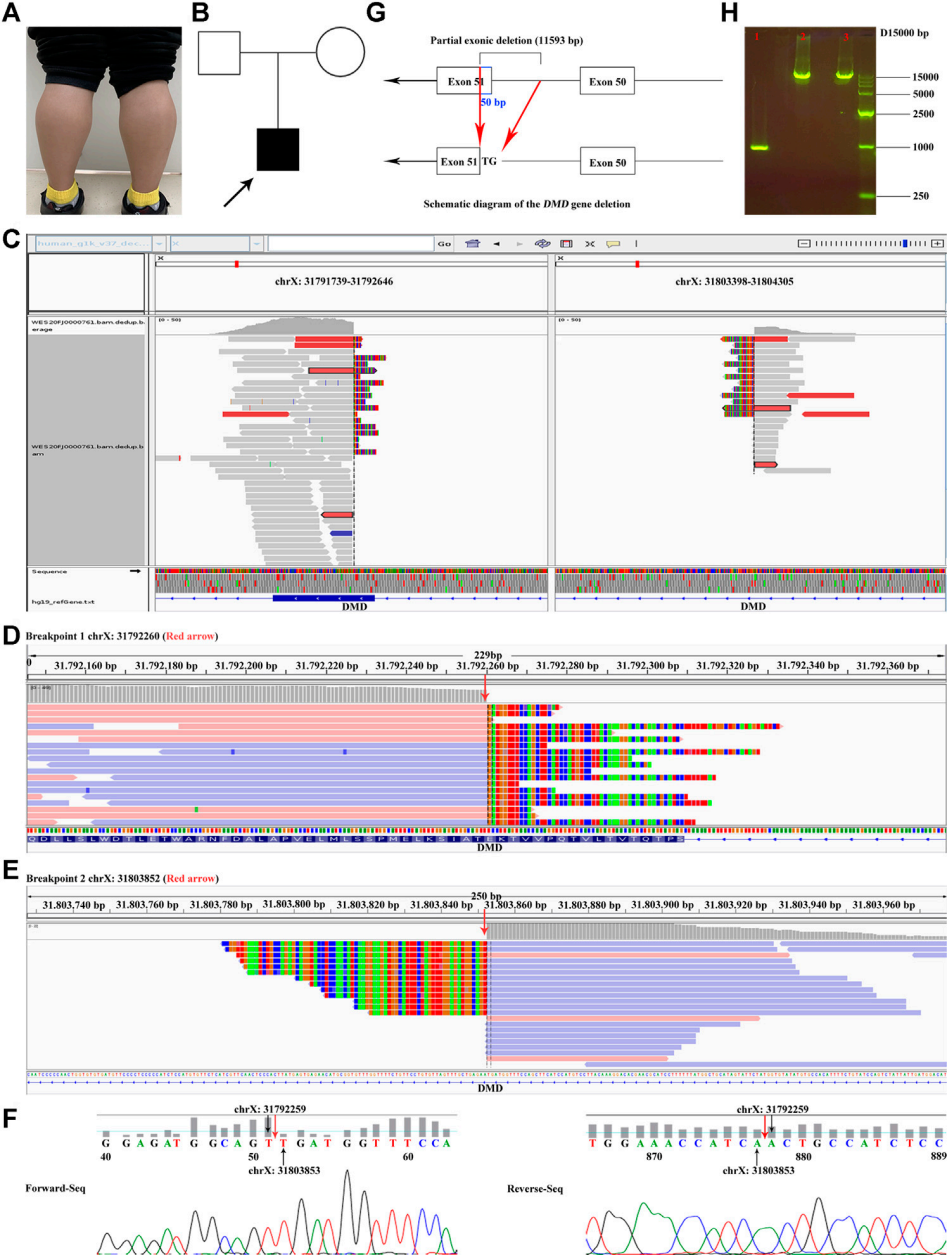
Proband clinical features, breakpoints suggested by WES, and breakpoints validation. **(A)** Bilateral gastrocnemius hypertrophy of the proband in November 2020. **(B)** Family tree. **(C)** The overview of the two breakpoints after analyzing the original BAM file of the proband. One breakpoint is in chrX:31791739–31792646; the other is in chrX:31803398–31804305. **(D, E)** The two breakpoints (red arrow) of *DMD* suggested by CNV and breakpoint analysis of the WES data. **(F)** Validation of the breakpoints by Sanger sequencing. Red arrow represents the positions of the breakpoints. **(G)** Diagrammatic sketch of the partial exonic deletion. **(H)** Results of agarose gel electrophoresis. 1. Proband; 2. Mother; 3. Negative control. The PCR product of the proband was approximately 1 kb, while those of the mother and the negative control were between 10 and 15 kb. Supplementary Videos. Various states of the proband: standing up after squatted down, going up and down stairs, and walking.

The proband was a 10-year-old boy who had developed more slowly than his peers since birth—for example, he was unable to walk until he was approximately 1.5 years old. As time progressed, both his calves became thick and hard (Figure 1A). He also struggled with walking, running, ascending stairs, squatting, and standing up. Because the proband’s family was living in a remote and underserved area, the proband remained undiagnosed until July 2020, when he was diagnosed with DMD in Henan Children’s Hospital. Electromyogram results confirmed myogenic damage of the nerves and muscles of both lower limbs and the left upper limb of the proband and the mother (Supplementary Table S2). The concentrations of creatine kinase (CK) and creatine kinase isoenzymes (CK-MB) in the venous serum of the proband were 14,984.0 U/L and 803.0 U/L (Supplementary Table S3), respectively. The proband had no family history of DMD (Figure 1B). Since the pathogenetic cause was not confirmed in Henan Children’s Hospital, the family came to our center in August 2020 to seek additional information about the genetic etiology of DMD.

### Deletions and Duplications in *DMD* of the Proband

MLPA analysis suggested that there was no deletion or duplication of exons in *DMD* (NM_004006.2) of the proband and the mother (Supplementary Figures S1A,B, S2A,B). Furthermore, the accuracy of kinship among the proband and the parents was confirmed by QF-PCR (Supplementary Table S4; Supplementary Figure S3). The quality control of the WES data is summarized in Supplementary Table S5. No candidate SNVs or small INDELs in *DMD* were found, and there was no significant variation in exon coverage. However, a 11,593-bp hemizygous deletion (c.7310-11543_7359del, chrX:g.31792260-31803852) spanning exon 51 and intron 50 in *DMD* was identified by CNV and breakpoint analysis of the WES data (Figure 1C–E). The 5′ breakpoint was only 50 bp from the 5′ end of exon 51 (Figure 1G).

### Additional Confirmation of the Breakpoints

The breakpoints of the large deletion were further confirmed by agarose gel electrophoresis and Sanger sequencing (Figure 1F). The results confirmed that the 5′ end 50 bp (chrX:31792260–31792309) in exon 51 and 3′ end 11,543 bp in intron 50 of *DMD* were hemizygous deletion in the proband. Furthermore, this deletion was *de novo*, since the PCR product of the proband was approximately 1 kb, and those of the mother and the negative control were between 10 and 15 kb (Figure 1H).

The quality control of the LR-WGS data is presented in Supplementary Tables S6–S8 and Supplementary Figure S4. A total of two reads captured the large deletions in *DMD*, one of ∼40 kb (read name: 97d7f30e-0f30-4ddc-b66d-c8c82c0fb221; chrX:31790731–31792260, chrX:31803853–31842466) and the other of ∼2.3 kb (read name: 1e46feed-d90e-44b8-bc47-56eb0495b76d; chrX:31791318–31792259, chrX:31803853–31805193) (Supplementary Figure S5, red arrows; Supplementary VCF File, line 1,569). The breakpoints of the large deletion were consistent with those identified by WES and Sanger sequencing. The structural variants (SVs; >1 kb) are listed in the Supplementary VCF File (GRCh37/hg19).

## Discussion

MLPA is often the first assay performed in DMD case studies, as deletion(s) and duplication(s) of one or more exons account for ∼67% of *DMD* variants. If the results of MLPA are negative, targeted NGS or WES will then be performed to further identify the genetic etiology of DMD (Aartsma-Rus et al., 2016). If the results are negative again, there are currently no prescriptive guidelines for further technical implementations. As a result, there are still undiagnosed cases of DMD. Most often, undiagnosed DMD cases are chimerism cases, rare mutation cases (e.g., gene inversion and translocation), and variable splicing mutation cases. Detection of deep intron or rare variant cases using LR-WGS will improve patient management and greatly contribute to the accuracy of prenatal diagnosis for mothers in the future.

In the present study, the MLPA assay was negative, indicating that no deletion or duplication of one or more exons was observed in either the proband or the mother (Supplementary Figures S1, S2; red arrow and red box), which may be due to that the probe of exon 51 for *DMD* (NM_004006.2; ligation site: 7,666–7,667; 24 nt adjacent to ligation site: GCT​CTG​GCA​GAT-TTC​AAC​CGG​GCT) was located on the upstream of the breakpoints with reference to the direction of the genome (GRCh37/hg19). Considering the high specificity of the clinical phenotype and other biochemical test results (Figure 1A; Supplementary Tables S2,S3; Supplementary Videos), the proband was still diagnosed with DMD. To verify the previous results and explore whether there is gene heterogeneity in the disease, WES was performed for the proband. The results of routine SNV analysis of the WES data were still negative; however, CNV and breakpoint analysis of the WES data suggested a possible out-of-frame deletion (representing most mutational events in patients with severe DMD) of c.7310-11543_7359del (chrX:g.31792260_31803852del) of *DMD* (Figure 1C–E). Moreover, this large intronic deletion may affect splicing and exon skipping of *DMD*. In addition, large intronic deletions may result in clinical effects due to splicing and exon skipping. The breakpoints of chrX:31792260 and chrX:31803852 were in exon 51 and intron 50, respectively (Figure 1G). This was confirmed by Sanger sequencing (Figure 1F). LR-WGS was needed to further explore whether there were more complex variations in the deep intron regions or intron–exon junction regions in *DMD* and to determine if the LR-WGS data collected (Supplementary Figure S5; Supplementary VCF File) were consistent with those of WES and Sanger sequencing. Lastly, although the large complex deletion of the proband was *de novo* in this study (Figure 1H), the prenatal diagnosis of *DMD* for the mother during pregnancy had to be performed since the possibility of gonadal chimerism could not be ruled out.

The genetic diagnosis in this case demonstrates a new application of WES-CNV analysis, and breakpoint analysis verified the accuracy of LR-WGS for one-time analysis of SVs. Although CNV and breakpoint analysis has not regularly been performed in conjunction with WES, CNVs can be potentially detected by WES (Nam et al., 2016). Recent attempts at optimizing the analytic pipeline for CNV detection have brought newfound attention to WES-CNV analysis (Zhao et al., 2020; Zhai et al., 2021).

The major advantage of LR-WGS is that it can directly analyze the SVs of DMD cases that produce negative WES results, without fragmentation of the whole genome of the target sample. The major disadvantage of LR-WGS is its relatively high cost: performing LR-WGS on an ONT sequencer is expensive and cannot be implemented in routine practice. Sanger sequencing can also detect breakpoints. Although Sanger sequencing does not give researchers as much specificity as LR-WGS does, it is much less expensive and more applicable for routine purposes. The use of LR-WGS for detecting intronic SVs and CNVs cannot be underestimated. In this study, it seems that ONT sequencing was not necessary when Sanger sequencing was sufficient to identify the deletion breakpoints. However, LR-WGS is carried out after Sanger sequencing for the aim described in the previous text. Moreover, this genetic diagnosis may not be suitable for the patient in this study. However, in the clinic, for patients with unremarkable results of MLPA and WES, ONT sequencing might be another selective method to determine the genetic etiology of rare genetic diseases, including DMD. This case may provide new data that will enhance the accessibility of research and development of *DMD* gene treatments for the partial deletion of exon 51.

A major limitation of this study was that RNA sequencing of a muscle biopsy sample from the proband was not performed to assess the overall clinical effect of this large deletion since it was extremely difficult to collect muscle biopsy sample from the proband.

In conclusion, this study reported a *de novo* partial exonic deletion in *DMD*. The discovery of this partial exonic deletion provided a theoretical basis for prenatal gene diagnosis for the mother in this family and will potentially help improve the efficiency of gene diagnosis.

## Data Availability

The datasets for this article are not publicly available due to concerns regarding participant/patient anonymity. Requests to access the datasets should be directed to the corresponding authors.
