# Supplementary material for: Novel Partial Exon 51 Deletion in the Duchenne Muscular Dystrophy Gene Identified via Whole Exome Sequencing and Long-Read Whole-Genome Sequencing
Source: Front Genet. 2021 Nov 26;12:762987. doi: 10.3389/fgene.2021.762987 (PMC8662377; doi:10.3389/fgene.2021.762987)
Supplement: Supplementary file 1 [file DataSheet1.ZIP › All the Supplementary Materials/Supplementary File 1.docx]

**Supplementary File 1. CNV and breakpoint analysis method of WES.**

Copy number variants (CNVs) were detected respectively by read depth (RD) and breakpoint (BP) signal using BAM file removed duplicate reads. The script calling CNVs was implemented in the R Programming Language.

First, read counts were calculated on each captured exon region, and the whole exome can be split into 237,217 regions with an average of 340 bp. All read counts were normalized to the same total read count as baseline data, which was created by 10 control male samples performed in the same pipeline. Read counts of baseline samples were already counted and normalized, and the region with a read count less than 10 was filtered. Poisson regression model was then fitted into all baseline count data and the Non-Linear Minimization method was used to estimate λ by each exon. The model:

$${RC}_{i}\sim Pois(\lambda_{i})$$

where RC was all read count and λ was the parameter of model on region i. Next, we used λ to calculate the probability of patient sample’s read count occurring in the fixed read count interval. Regions with probability over a threshold (*p*≤${1E}^{-4}$) were probable CNV regions and the continuous filtered region were reduced. Finally, relative copy numbers of region or continuous regions, i.e. exon or continuous exons were calculated by patient and baseline samples:

$$\mathrm{cn}_{j}= 2^log2(\frac{{rc}_{j}}{{bc}_{j}})$$

Where *rc* was patient’s read count and *bc* were baseline samples’ median read count on CNV region *j*, and cn *j* was relative copy number of CNV *j*.

In order to detect CNVs near the end of exons, which had insignificantly variation on depth, we also called breakpoints near exons. CNVs, ones of structure variants (SVs), often had two breakpoints on genome, each might cause soft clipped alignment, a symbol of SVs. The soft-clipped reads were identified by ‘S’ CICAR tags in the BAM file.

First, we extracted all soft-clipped reads and split them into map and soft clips. Then two kinds of clips that from reads mapping on the same position of reference, would be respectively assembled to paired-long sequence and aligned to the reference. Next, we identified the deletion using the alignment position and orientation of pair clips. If pair clips had the same alignment orientation and their interval was over 50 bp, the region they mapped was probably occurred a deletion if over three soft-clipped reads supported this breakpoint. Finally, the results of CNV and breakpoint analysis were merged and annotated with gene and transcript information.
