# Supplementary material for: Novel Partial Exon 51 Deletion in the Duchenne Muscular Dystrophy Gene Identified via Whole Exome Sequencing and Long-Read Whole-Genome Sequencing
Source: Front Genet. 2021 Nov 26;12:762987. doi: 10.3389/fgene.2021.762987 (PMC8662377; doi:10.3389/fgene.2021.762987)

**Supplementary Table 1. Primers for Sanger sequencing.**

| **Primer ID** | **Primer sequence** | **Location (hg19/GRCh37)** | **PCR product of wild type** | **PCR product of mutant type** |
| --- | --- | --- | --- | --- |
| *DMD*-Breakpoint-F | CAAGCCCGGTTGAAATCTGC | chrX:31792183-31792202 | 12574 bp | 983 bp |
| *DMD*-Breakpoint-R | TTGGTAGTGTGGGTGCATCG | chrX:31804738-31804757 |  |  |

**Supplementary Table 2. EMG data of the proband.**

| **Motor nerve conduction velocity (Nerve)** | **Incubation period (ms)** | **Amplitude (mV)** | **Distance (mm)** | **Velocity (m/s)** |
| --- | --- | --- | --- | --- |
| Ulnar nerve motor left |  |  |  |  |
| Wrist - ADM | 2.32 | 10.50 |  |  |
| Elbow - wrist | 6.01 | 10.00 | 205 | 55.6 |
| Median nerve motor left |  |  |  |  |
| Wrist - APB | 2.61 | 12.1 |  |  |
| Elbow - wrist | 6.96 | 11.7 | 245 | 56.3 |
| Eyelid nerve motor left |  |  |  |  |
| Erb - musculus biceps brachii | 3.50 | 4.60 |  |  |
| Femoral nerve motor left |  |  |  |  |
| Groin - Rectus femoris | 2.58 | 5.00 ↓↓↓ |  |  |
| Femoral nerve motor right |  |  |  |  |
| Groin - Rectus femoris | 3.13 | 3.80 ↓↓↓ |  |  |
| Tibial nerve motor left |  |  |  |  |
| Ankle - AH | 2.81 | 24.80 |  |  |
| Knee - Ankle | 8.94 | 23.4 | 330 | 53.8 |
| Tibial nerve motor right |  |  |  |  |
| Ankle - AH | 2.54 | 22.0 |  |  |
| Knee - Ankle | 8.89 | 20.4 | 330 | 52.0 |
| Nervus peroneus communis motor left |  |  |  |  |
| Ankle - EDB | 3.01 | 4.10 |  |  |
| Fibular head - Ankle | 8.47 | 4.10 | 300 | 54.9 |
| Nervus peroneus communis motor right |  |  |  |  |
| Ankle - EDB | 2.90 | 3.40 |  |  |
| Fibular head - Ankle | 7.80 | 3.00 ↓ | 260 | 53.10 |
| **Sensory nerve conduction velocity (Nerve)** | **Incubation period (ms)** | **Amplitude (mV)** | **Distance (mm)** | **Velocity (m/s)** |
| Ulnar nerve sense left |  |  |  |  |
| Finger -Ⅴ Twist | 3.04 | 36.50 | 100 | 32.90 |
| Radial nerve Sural nerve |  |  |  |  |
| EPL tendon - Twist | 2.48 | 18.9 | 78.00 | 31.5 |
| Median nerve sense left |  |  |  |  |
| Finger -Ⅱ Twist | 2.56 | 89.90 | 100 | 39.10 |
| Sural nerve sense left |  |  |  |  |
| Middle calf - External Malleolus | 2.59 | 24.40 | 100 | 38.60 |
| Sural nerve sense right |  |  |  |  |
| Middle calf - External Malleolus | 2.56 | 14.10 | 90.00 | 35.20 |

**F wave**

| **F wave** | **M-Lat (ms)** | **M-Lat Min (ms)** | **F (#)** | **F% (F%)** | **Dist (mm)** | **CV (m/s)** |
| --- | --- | --- | --- | --- | --- | --- |
| Median nerve F-wave left |  |  |  |  |  |  |
| Twist - APB | 2.40 | 21.70 | 3.00 | 100 | 580 | 63.40 |
| Tibial nerve F-wave left |  |  |  |  |  |  |
| Ankle - AH | 1.89 | 36.20 | 3.00 | 100 | 835 | 50.10 |
| Tibial nerve F-wave left |  |  |  |  |  |  |
| Ankle - AH | 2.50 | 36.40 | 3.00 | 100 | 845 | 51.40 |

**H reflex**

| **H reflex** | **M-Lat (ms)** | **H-Lat (ms)** | **M-amp (mv)** | **H-amp (mv)** | **M/H** |
| --- | --- | --- | --- | --- | --- |
| Tibial nerve H reflex left |  |  |  |  |  |
| Knee - gastrocnemius | 3.60 | 22.90 | 3.70 | 1.74 | 0.46 |
| Tibial nerve H reflex right |  |  |  |  |  |
| Knee - gastrocnemius | 4.20 | 22.90 | 0.85 | 2.30 | 2.70 |

**EMG with concentric needle**

|  | **Spontaneous potential** | | | | **Voltage (uV)** | | **Average time limit (ms)** | **Increase decrease (%)** | **Polyphase wave** | **Motion waveform of heavy contraction** | **Peak voltage (mV)** |
| --- | --- | --- | --- | --- | --- | --- | --- | --- | --- | --- | --- |
|  | **Insert** | **Fibrillation** | **Normal Phase** | **Others** | | **754** | **7.0** | **↓ 25** | **78.9** | **Pathological interference phase** | **0.794** |
| Left Biceps (medial head) | - | + | + | - | | 685 | 7.1 | ↓ 24 | 75.0 | Pathological interference phase | 0.737 |
| Left Quadriceps femoris medial head | - | - | - | - | | 931 | 6.6 | ↓ 29 | 70.0 | Pathological interference phase | 0.799 |
| Right Quadriceps femoris medial head | - | - | - | - | | 723 | 8.5 | ↓ 24 | 100 | Pathological interference phase | 1.243 |
| Left Tibialis anterior medial head | - | - | - | - | | 659 | 7.8 | ↓ 30 | 93.3 | Pure mixed phase | 1.254 |

**Supplementary Table 3. Serology detection of the proband.**

| **Abbreviation** | **Project name** | **Results** | **Reference range** | **Units** |
| --- | --- | --- | --- | --- |
| TBIL | Total bilirubin | 7.7 | 0-20 | μmol/L |
| DBIL | Direct bilirubin | 3.3 | 0-7 | μmol/L |
| IBIL | Indirect bilirubin | 4.4 | 0-17 | μmol/L |
| ALT | Alanine aminotransferase | 355.2 ↑ | 0-40 | U/L |
| AST | Aspartate aminotransferase | 202.8 ↑ | 0-40 | U/L |
| ALP | Alkaline phosphatase | 126.7 | 12-300 | U/L |
| GGT | γ-glutamyl transpeptidase | 9.2 | 0-50 | U/L |
| TP | Total protein | 72.3 | 60-85 | g/L |
| ALB | Albumin | 47.8 | 35-55 | g/L |
| GLOB | Globulin | 24.5 | 20-45 | g/L |
| LDH | Lactate dehydrogenase | 1058.0 ↑ | 80-245 | U/L |
| HBDH | α-Hydroxybutyrate dehydrogenase | 803.0 ↑ | 80-200 | U/L |
| CK | Creatine kinase | 14984.0 ↑ | 25-200 | U/L |
| CK-MB | Creatine kinase isoenzymes | 225.0 ↑ | 0-24 | U/L |
| UREA | Urea | 3.4 | 1.7-7.1 | mmol/L |
| CREA | Creatinine | 22.2 | 18-80 | μmol/L |
| UA | Uric acid | 225.2 | 134-415 | μmol/L |
| K | Potassium | 3.90 | 3.5-5.5 | mmol/L |
| NA | Sodium | 137.6 | 135-145 | mmol/L |
| CL | Chlorine | 103.9 | 96-108 | mmol/L |
| CA | Calcium | 2.39 | 2.1-2.75 | mmol/L |
| P | Phosphorus | 1.60 | 1.29-1.94 | mmol/L |
| Mg | Magnesium | 1.30 | 0.62-1.3 | mmol/L |

**Supplementary Table 4. STR loci of the proband and the parents.**

| **STR locus** | **Mother** | | **Proband** | | **Father** | | **Father gene 1** | **Father gene 2** | **Calculation formula** | **PI** |
| --- | --- | --- | --- | --- | --- | --- | --- | --- | --- | --- |
| D19S433 | 14 | 14 | 14 | 15 | 15 | 15.2 | 0.0594 |  | 1/(2*p) | 8.4175 |
| D5S818 | 10 | 11 | 11 | 12 | 11 | 12 | 0.2406 |  | 1/(2*p) | 2.0781 |
| D21S11 | 29 | 33.2 | 32 | 33.2 | 30 | 32 | 0.0285 |  | 1/(2*p) | 17.5439 |
| D18S51 | 13 | 19 | 13 | 14 | 14 | 15 | 0.2160 |  | 1/(2*p) | 2.3148 |
| D6S1043 | 10 | 18 | 18 | 18 | 18 | 18 | 0.1719 |  | 1/p | 5.8173 |
| D3S1358 | 15 | 15 | 15 | 15 | 15 | 15 | 0.3453 |  | 1/p | 2.8960 |
| D13S317 | 8 | 8 | 8 | 11 | 11 | 12 | 0.2368 |  | 1/(2*p) | 2.1115 |
| D7S820 | 8 | 9 | 8 | 11 | 11 | 12 | 0.3471 |  | 1/(2*p) | 1.4405 |
| D16S539 | 10 | 10 | 9 | 10 | 9 | 9 | 0.2840 |  | 1/p | 3.5211 |
| CSF1PO | 12 | 12 | 10 | 12 | 9 | 10 | 0.2433 |  | 1/(2*p) | 2.0551 |
| Penta D | 8 | 9 | 8 | 12 | 12 | 12 | 0.1328 |  | 1/p | 7.5301 |
| AMEL | 0 | 0 | 0 | 1 | 0 | 1 |  |  |  |  |
| vWA | 17 | 18 | 13 | 18 | 13 | 13 | 0.0020 |  | 1/p | 500.0000 |
| D8S1179 | 14 | 16 | 14 | 16 | 12 | 14 | 0.1852 | 0.0737 | 1/[2*(p+q)] | 1.9312 |
| TPOX | 11 | 12 | 9 | 11 | 8 | 9 | 0.1300 |  | 1/(2*p) | 3.8462 |
| Penta E | 15 | 19 | 15 | 15 | 15 | 15 | 0.0752 |  | 1/p | 13.2979 |
| TH01 | 7 | 10 | 7 | 9 | 9 | 9 | 0.5215 |  | 1/p | 1.9175 |
| D12S391 | 20 | 20 | 20 | 21 | 15 | 21 | 0.1250 |  | 1/(2*p) | 4.0000 |
| D2S1338 | 19 | 23 | 19 | 23 | 19 | 23 | 0.1969 | 0.2031 | 1/(p+q) | 2.5000 |
| FGA | 24 | 25 | 22 | 24 | 22 | 22 | 0.1866 |  | 1/p | 5.3591 |
|  |  |  |  |  |  |  |  | PI (Accumulation) 10066833320740.5140 | | |
|  |  |  |  |  |  |  |  | RCP 1.0000000000 | | |

**Supplementary Table 5. Quality control of the WES data of the proband.**

| **20×Coverage** | **Data size (Gb)** | **Capture efficiency (%)** | **Duplication rate (%)** | **Ave. sequencing depth (×)** |
| --- | --- | --- | --- | --- |
| 98.13% | 11.6 | 66 | 16.65 | 110 |

**Supplementary Table 6. Summary of read alignments and SV discovery of LR-WGS .**

Executive Summary

| **Primary alignments** | **Ave. read quality** | **Ave. read length** | **Variants found** |
| --- | --- | --- | --- |
| 1.9 M | 13.3 | 3.5 kb | 894.0 |

### Supplementary Table 7. Read alignments of LR-WGS.

Summary of read alignments to the reference genome. Note that read-based statistics (e.g Ave. read length) are not reported for secondary and supplementary alignments where they would otherwise overcount.

| **Feature** | **Primary** | **Secondary** | **Supplementary** |
| --- | --- | --- | --- |
| Alignments | 1941317 | 685926 | 114469 |
| Unique Reads | 1941317 | 206960 | 92532 |
| Ave. read length | 3417 | - | - |
| Ave. read quality | 13.255 | - | - |
| Ave. mapping quality | 60 | 0 | 0 |
| Ave. align length | 3325 | 2990 | 1966 |
| Total aligned bases | 12287067969 | 3663639524 | 378995992 |
| Total clipped bases | 551014332 | 91902114 | 1389306844 |

**Supplementary Table 8. Structural Variants of LR-WGS.**

The structural variants (SVs) found by the analysis and reported in the output .vcf are summarized below.

| **SVs Type** | **Deletion** | **Duplication** | **Insertion** |
| --- | --- | --- | --- |
| **Count** | 469 | 4 | 421 |
| Min. Length | 51 | 481 | 51 |
| Ave. Length | 161 | 3677.5 | 134 |
| Max. Length | 142142 | 4972 | 1986 |

**Supplementary Figure 1A. MLPA analysis of the proband (P034).**

**
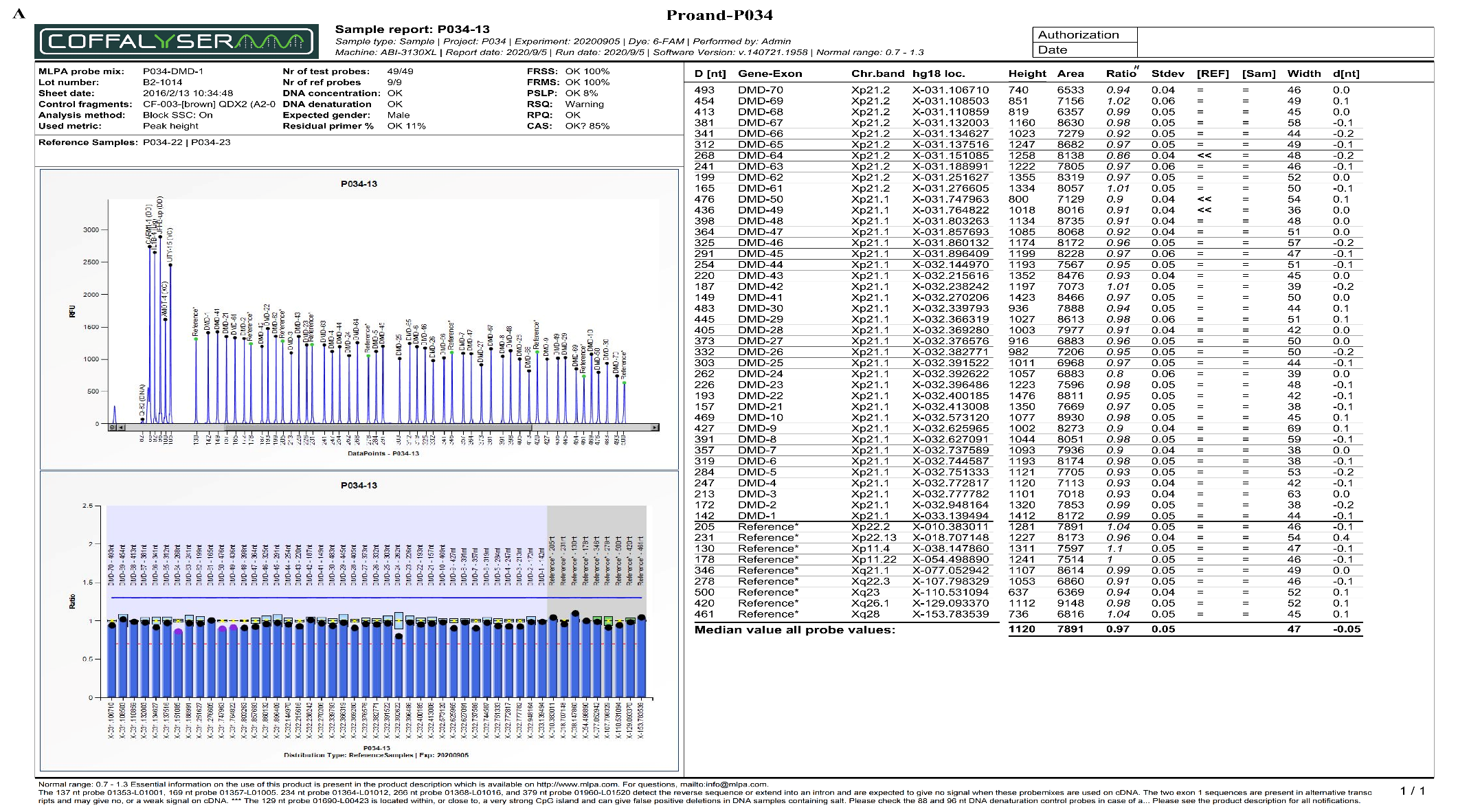
**

**Supplementary Figure 1B. MLPA analysis of the proband (P035).**

**
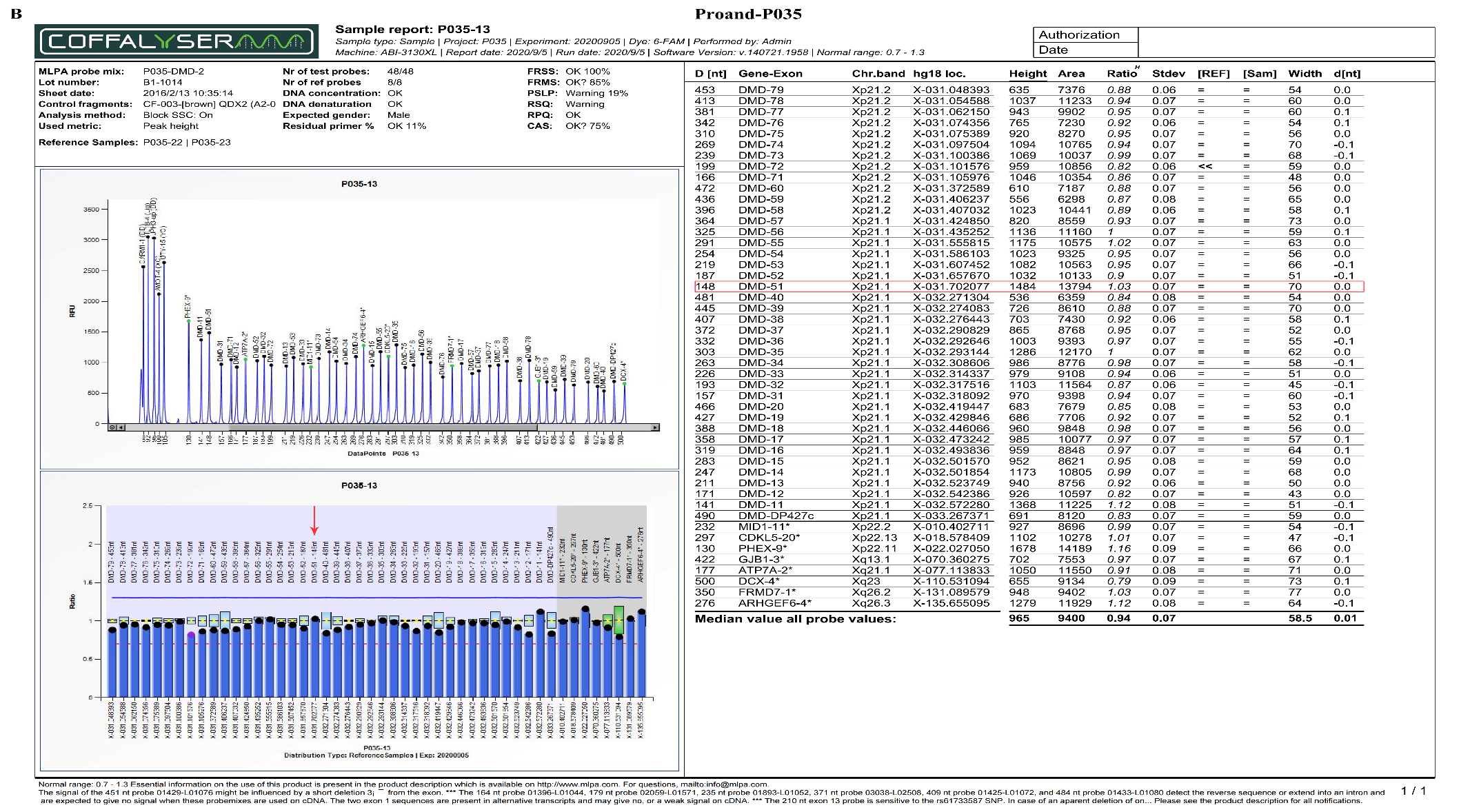
**

**Supplementary Figure 2A. MLPA analysis of the mother (P034).**

**
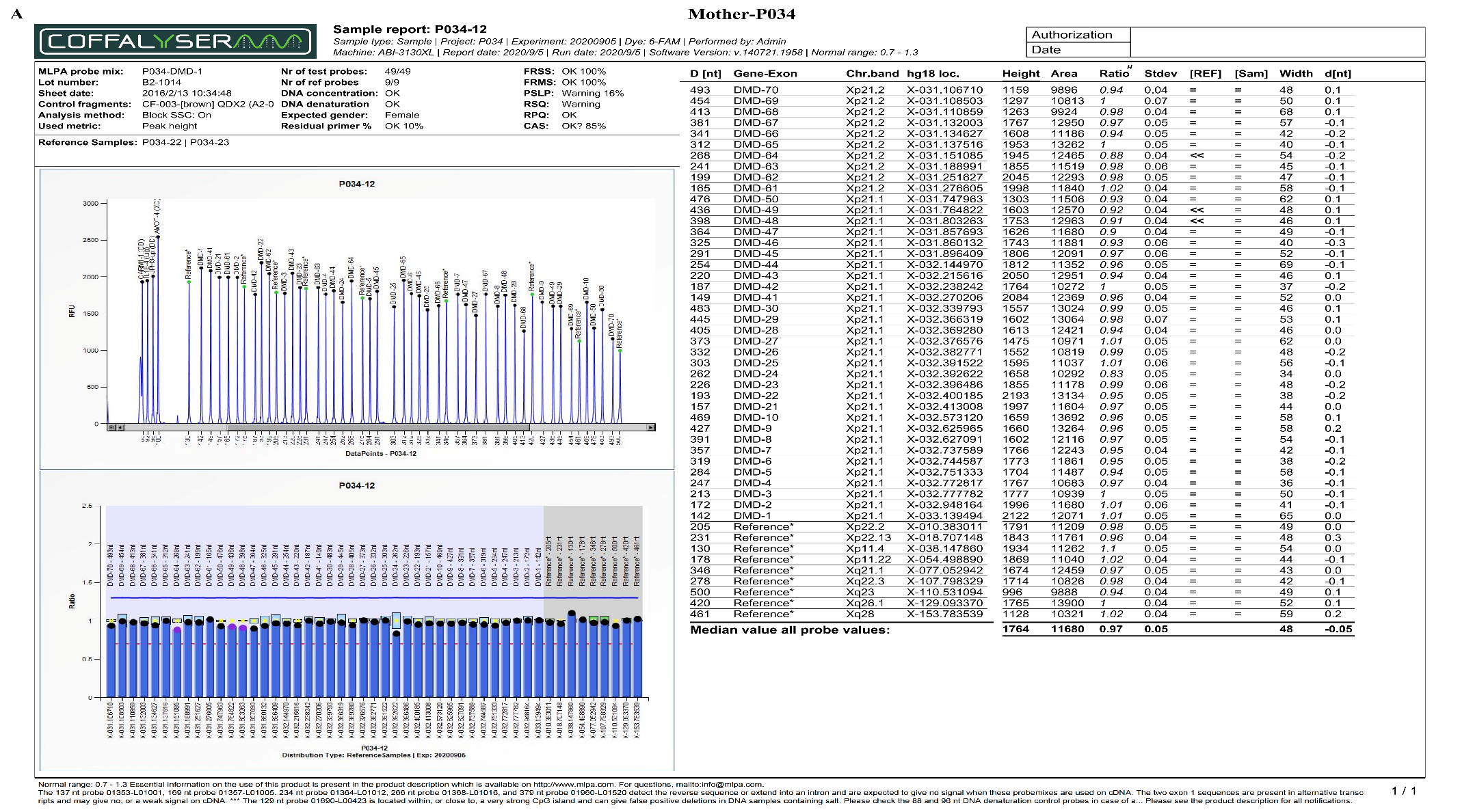
**

**Supplementary Figure 2B. MLPA analysis of the mother (P035).**

**
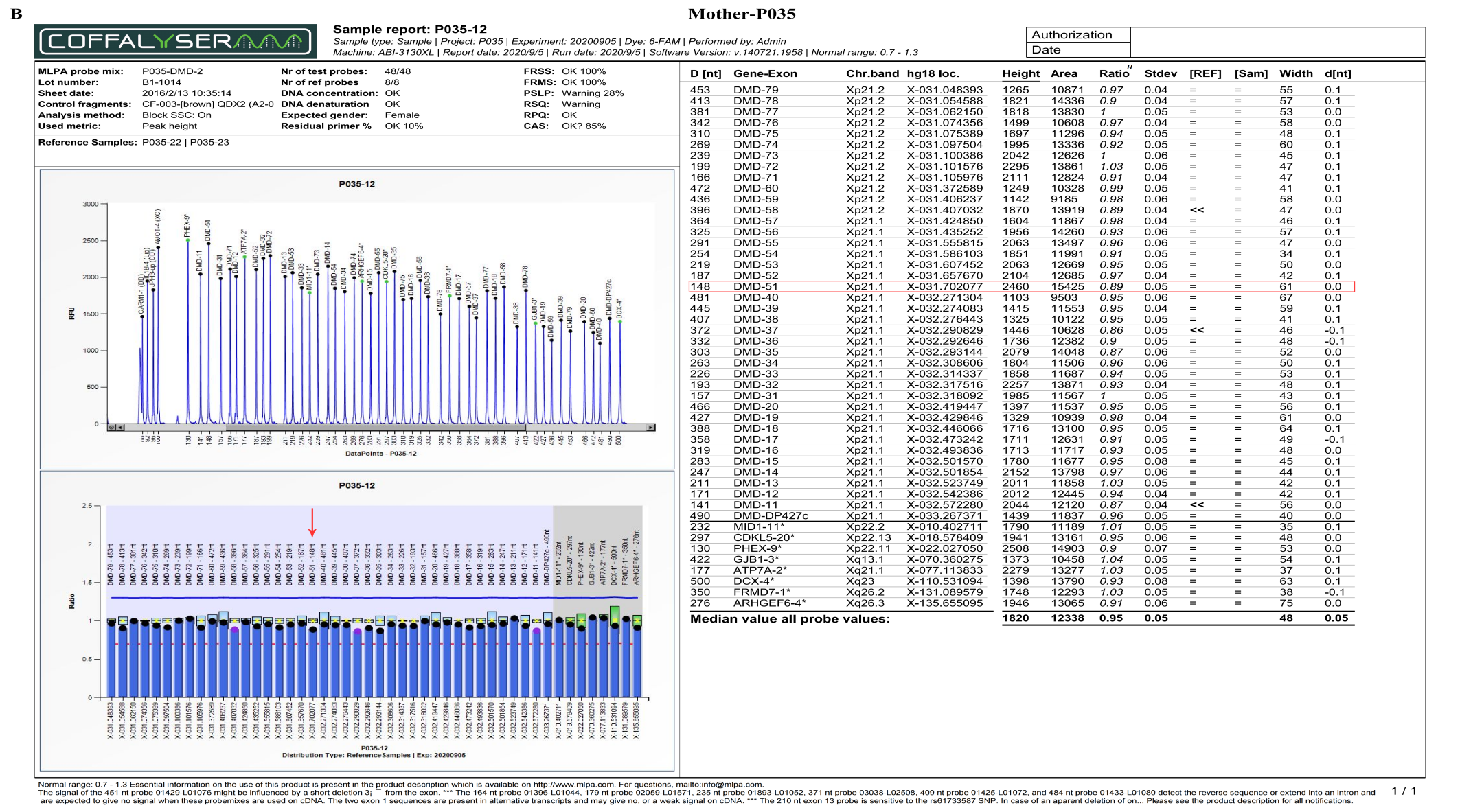
**

**Supplementary Figure 3. QF-PCR results of the proband and the parents.**


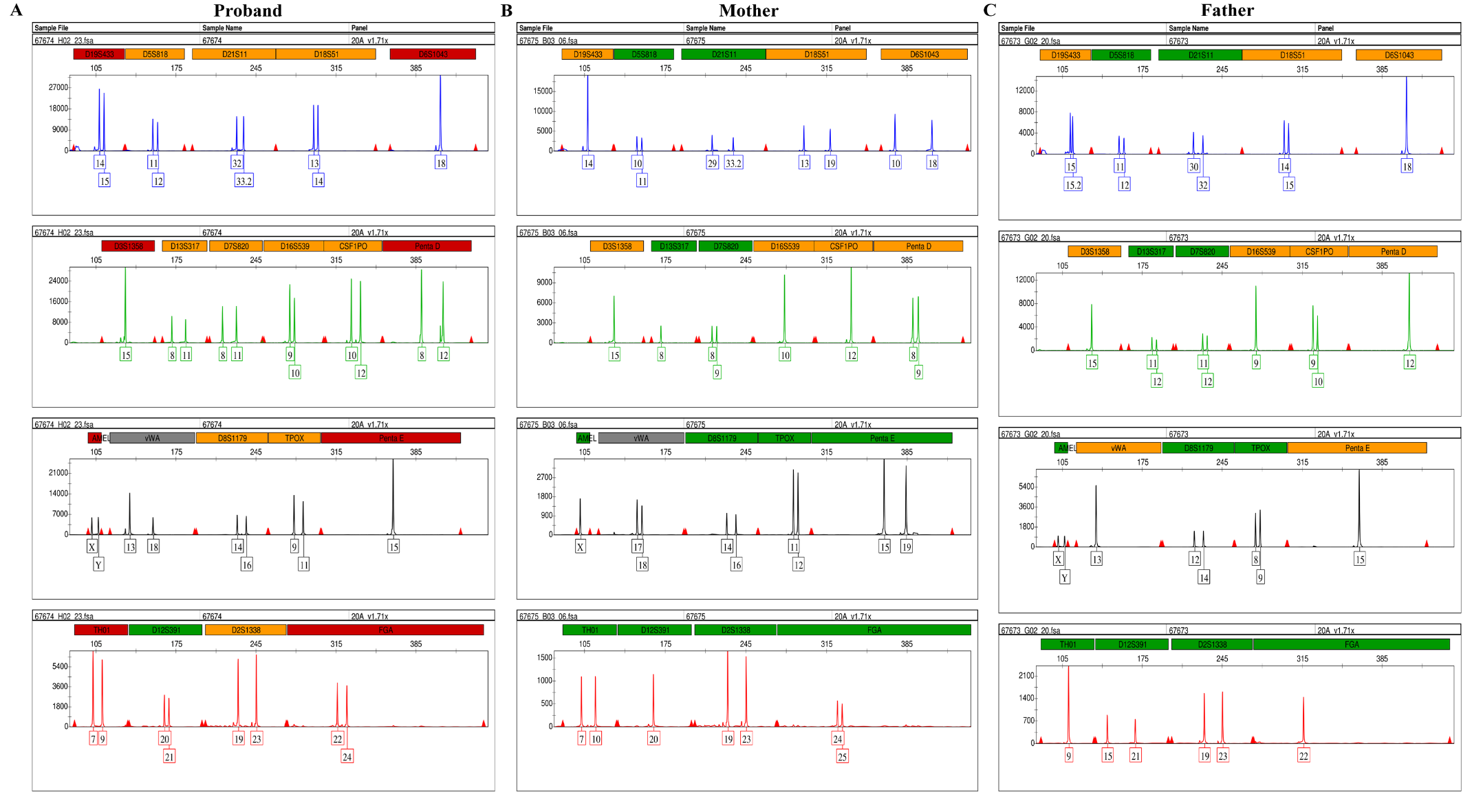


### Supplementary Figure 4. Primary alignment statistics of LR-WGS.

The following plots give an overall impression of read quality. Accuracy should show a strong peak above 93% with alignment containment above 98%.


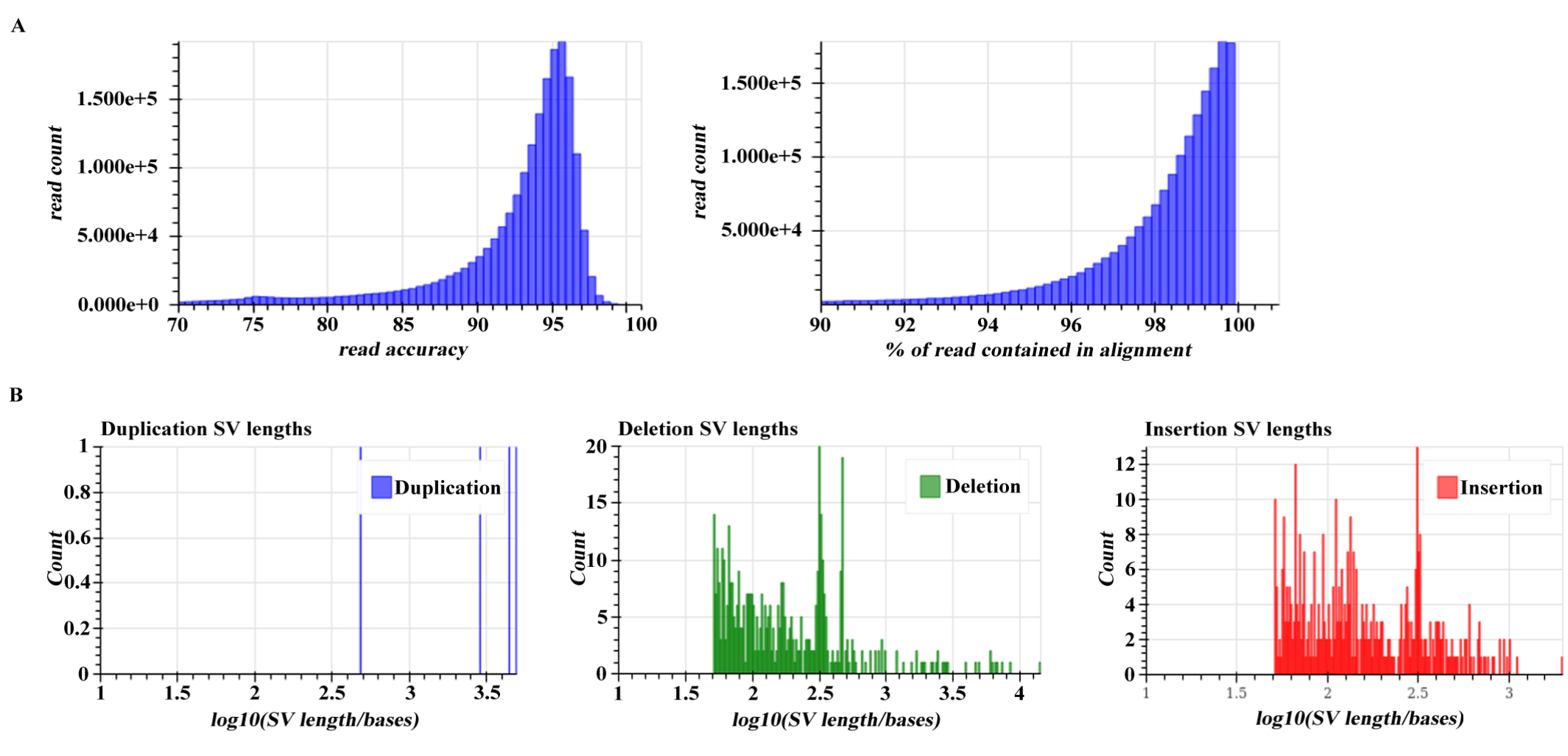


### Supplementary Figure 5. Two long reads in *DMD* captured by LR-WGS (red arrows).

One is ~40 kb (chrX:31790731-31792260, chrX:31803853-31842466), and the other is ~2.3 kb (chrX:31791318-31792259, chrX:31803853- 31805193).


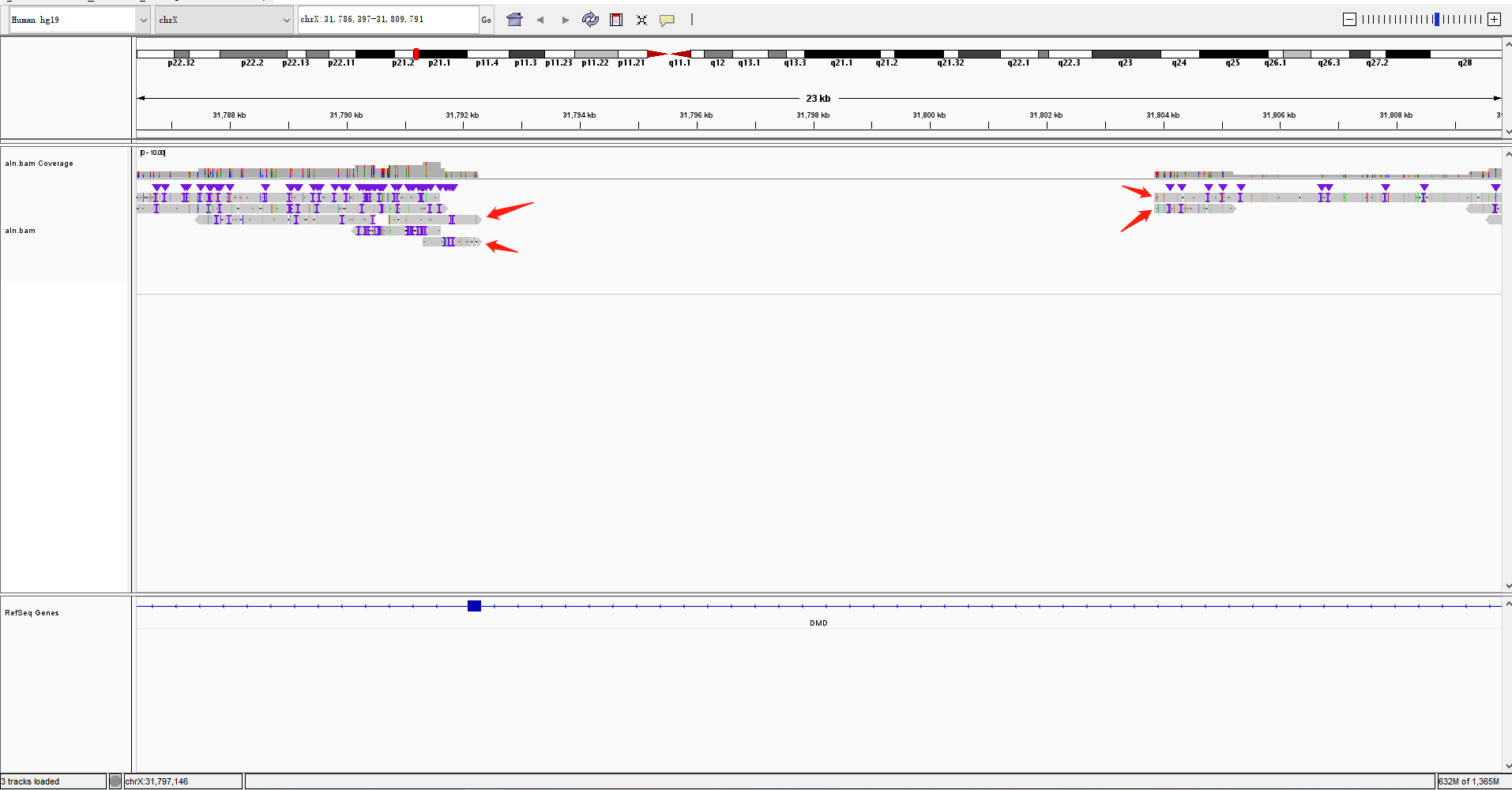

Supplement: Supplementary file 1 [file DataSheet1.ZIP › All the Supplementary Materials/Supplementary Tables and Figures-2021-10-26.docx]
